# Impact of the NK Cell Receptor LIR-1 (ILT-2/CD85j/LILRB1) on Cytotoxicity against Multiple Myeloma

**DOI:** 10.1155/2012/652130

**Published:** 2012-07-10

**Authors:** Silke Heidenreich, Christine zu Eulenburg, York Hildebrandt, Thomas Stübig, Heidi Sierich, Anita Badbaran, Thomas H. Eiermann, Thomas M. C. Binder, Nicolaus Kröger

**Affiliations:** ^1^Clinic for Stem Cell Transplantation, University Medical Center Hamburg-Eppendorf, Martinistrasse 52, 20246 Hamburg, Germany; ^2^Department of Medical Biometry and Epidemiology, University Medical Center Hamburg-Eppendorf, Hamburg, Germany; ^3^Department of Transfusion Medicine, University Medical Center Hamburg-Eppendorf, Hamburg, Germany

## Abstract

The role of different receptors in natural-killer- (NK-) cell-mediated cytotoxicity against multiple myeloma (MM) cells is unknown. We investigated if an enhancement of NK-cell-mediated cytotoxicity against MM could be reached by blocking of the inhibitory leukocyte immunoglobulin-like receptor 1 (LIR-1). Our investigations revealed high levels of LIR-1 expression not only on the NK cell line NK-92, but also on myeloma cells (MOLP-8, RPMI8226) as well as on a lymphoblastoid cell line (LBCL; IM-9). Subsequent cytotoxicity assays were designed to show the isolated effects of LIR-1 blocking on either the effector or the tumor side to rule out receptor-receptor interactions. Although NK-92 was shown to be capable of myeloma cell lysis, inhibition of LIR-1 on NK-92 did not enhance cytotoxicity. Targeting the receptor on MM and LBCL did not also alter NK-92-mediated lysis. We come to the conclusion that LIR-1 alone does not directly influence NK-cell-mediated cytotoxicity against myeloma. To our knowledge, this work provides the first investigation of the inhibitory capability of LIR-1 in NK-92-mediated cytotoxicity against MM and the first functional evaluation of LIR-1 on MM and LBCL.

## 1. Introduction

Understanding of NK cell function has undergone a long process since their identification in 1975 [1]. NK cells have initially been regarded as part of the innate immune system, not allowing any modulation of action with respect to their changing microenvironment. Their pattern of inhibitory and activating receptors was considered to be sufficient to adequately detect tumor cells by the lack of human leukocyte antigen (HLA) class I molecules. Those tumor cells were killed instantly and without any obvious need of coactivation by other cells of the immune system [2]. This unique feature among lymphocytes has now been understood to be only the basic function of response, which is completed by diverse interactions with especially dendritic cells (DC) and T cells [3]. NK cells do extensively communicate with their surroundings, and their still-not-fully-deciphered set of receptors detects changes in the normal surface pattern on all types of tissues.

NK cell receptors are functionally divided into activating and inhibitory receptors. Their main ligands are major histocompatibility complex I (MHC-I) molecules, while some of the receptors can directly recognize specific antigens on bacteria or damaged cells. Mainly three different subclasses of NK-cell receptors (NKRs) can be distinguished.

LIR and killer immunoglobulin-like receptors (KIRs) are type I transmembrane proteins of the immunoglobulin-like receptor superfamily (IgSF). Both recognize classical HLA class I molecules, while LIR can also interact with nonclassical HLA class I and bacteria with low binding affinities [2, 4–6]. The second group of natural cytotoxicity receptors (NCRs) also belongs to type I transmembrane proteins but has poorly defined ligands. Type II transmembrane proteins of the C-lectin type superfamily include natural killer cell lectin-like receptor group 2 (NKG2) receptors that form heterodimers with CD94 [2].

LIRs are expressed on subsets of NK cells and T cells, as well as on monocytes, B cells, and DC, with the widest distribution for LIR-1 [7–10].

LIR-1 is an inhibitory receptor also known as immunoglobulin-like transcript 2 (ILT-2)/CD85j or leukocyte immunoglobulin-like receptor, subfamily B member 1 (LILRB1) [7]. It has first been detected in searching for the counterpart of UL18, a cytomegalovirus encoded HLA class I homolog that is expressed on infected cells [8, 11, 12].

MM is an incurable disease that is characterized by the clonal proliferation of terminally differentiated plasma cells [13, 14]. Stem cell transplantation (SCT) is so far the only option to achieve long time remission of the disease [15]. To improve the outcome of MM patients, approaches like immunomodulation and cellular therapy are under investigation. NK cells are an attractive candidate for immune therapy. They kill tumor cells without antigen-specific priming [2] and are the the predominant lymphocyte subset within the first 90 days after transplantation [16–19]. LIR-1 is one of the main inhibitory NK cell receptors in this early phase after SCT [10, 16, 20].

 We therefore investigated the influence of LIR-1 on myeloma defeat. Hereby, we studied the effects of LIR-1 blocking of NK-92 as well as on a panel of tumor cell lines including MM. To our knowledge, these experiments provide the first data concerning the influence of isolated LIR-1 inhibition on NK cells with respect to myeloma cell lysis. Moreover, they provide the first functional study of LIR-1 on MM and on other tumor entities, taking into account its broad distribution among tissues.

## 2. Material and Methods

### 2.1. Cells

Unless otherwise stated, all media and supplements were obtained from Life Technologies. Natural killer cell line NK-92 was cultured in alpha-MEM supplemented with Earl's Salts and L-Glutamine, 12.5% equine serum, 12.5% fetal calf serum, 0.2 mM inositol (Sigma-Aldrich), 0.1 mM 2-mercaptoethanol (Sigma-Aldrich), 0.02 mM folic acid (Sigma-Aldrich), and 1% PenStrep. Cells were splitted every third day and received 200 U/mL rhIL-2 (CellSystems) with the fresh medium. Myeloma cell line MOLP-8 was cultured in RPMI1640 with 20% FCS and 1% PenStrep while IM-9, RPMI 8226, HL60, and K562 received the same medium and antibiotics but only 10% FCS. COS-7 cells were cultured in DMEM with 10% FCS and 1% PenStrep. JEG-3 was grown in Ham's F12 with 10% FCS and 1% PenStrep.

### 2.2. Flow Cytometry

Monoclonal antibodies (mAb) were phycoerythrin- (PE-) conjugated CD2 (RPA-2.10, BDPharmingen), CD159a (Z199, Beckman Coulter), CD85j (HP-F1, Beckman Coulter); Pacific Blue-conjugated CD16 (MOPC-21, BD Pharmingen); fluorescein isothiocyanate- (FITC-) conjugated CD25 (B1.49.9, Beckman Coulter) and anti-IgG (goat polyclonal anti-mouse IgG, Abcam); allophycocyanin (APC)-stained CD56 (B159, BD Pharmingen) as well as appropriate isotype controls. Unconjugated anti-HLA-I (HP-1F7) was obtained from Santa Cruz, anti-HLA-G (MEM-G/09) and -E (MEM-E/08) were obtained from Abcam. 7-Amino-Actinomycin D (7AAD, BD Pharmingen) was used to analyze dead cells. 20 *μ*L Anti-A, B reagent was used in each sample to block unspecific bindings (Ortho-Clinical Diagnostics). Cells were incubated with the antibody or isotype control for 30 minutes at 4°C, washed with PBS and, if appropriate, stained with a secondary antibody followed by an additional washing step. All samples were additionally stained with 7AAD. Fluorescence was measured on a BD FACSCanto II flow cytometer and BD FACSDIVA Software v.6.1.3 (Becton Dickinson) was used for data analysis.

### 2.3. Transfection of COS-7 Cells with LIR-1

As a positive control for western blot experiments, COS-7 cells were transfected with pCMV6-AC vector encoding for LIR-1 (OriGene) or pCMV6-XL5 as a mock control (OriGene), using FuGene HD Transfection Reagent (Promega) [21, 22]. All steps were performed according to the manufacturer's instructions. The vector was multiplied by transformation of E.coli cells (One Shot TOP10/P3 competent cells) with subsequent purification of the plasmids (Qiagen EndoFree Plasmid Maxi Kit).

### 2.4. Western Blot Analysis

Western blot was used to analyze expressed surface molecules of effector and target cells [23, 24]. Proteins were separated by sodium dodecyl sulfate polyacrylamide gel electrophoresis (SDS-PAGE) under reducing conditions. Gels (Life Technologies) were blotted onto nitrocellulose membranes (Whatman). Membranes were blocked for one hour with TBS-T buffer (0.05 M Tris-HCL, 0.15 M sodium chloride, 0.1% Tween 20) containing 3% nonfat dry milk. Incubation with the anti-LIR-1 (VMP55, Santa Cruz) was done overnight at 4°C under gentle agitation. After extensive washing with TBS-T, secondary one-hour incubation with horseradish-peroxidase-conjugated goat anti-mouse IgG (R&D Systems) was completed with additional washing. Membranes were stained with enhanced chemiluminescence agent (GE Healthcare) and exposed to X-ray film (GE Healthcare). To confirm equal loading of all gel chambers, membranes were stripped from the specific antibody using Re-Blot solution (Millipore), followed by additional staining of *β*-Actin (ACTB, C4, Santa Cruz).

### 2.5. Cytotoxicity Assays

Cytolysis was determined in 4-hour chromium-release assays (CRAs) according to standard protocols [25–27]. Briefly, NK-92 and target cells were seeded out in fresh medium one day before functional assays. The next day target cells were labeled with 100 *μ*Ci sodium-51-chromate (51Cr) for 1.5 hours at 37°C in a humidified incubator with 5% CO_2_. Two washing steps were performed with PBS (Life Technologies) and assay medium (RPMI1640, 10% FCS, 1% PenStrep; Life Technologies), respectively. Cells were resuspended to a dilution of 5 × 10^3^ cells/100 *μ*L. NK-92 and tumor cells were coincubated at various effector : target (E : T) ratios in U-bottom microtiter plates at a total of 200 *μ*L assay medium. Maximum lysis or spontaneous release (SR) of 51Cr were induced by adding 5% Triton-X or assay medium to 100 *μ*L target cells, respectively. All samples were plated out in triplicates. Prior to the 4-hour incubation period, plates were carefully centrifuged to facilitate E : T contact. After incubation and additional centrifugation, 25 *μ*L supernatant were transferred to a 96-well plate (Isoplate 96, PerkinElmer). To each well, 150 *μ*L scintillation liquid were added (Rotiszint eco plus, Carl Roth). Plates were closed with Viewseal foils (Greiner Bio-One). Suspension was mixed thoroughly for 15 minutes at 19°C on an Eppendorf thermomixer and then measured at Wallac Trilux 1450 Microbeta Counter, Windows WS V. 2.70.004, PerkinElmer). The percentage of specific lysis was calculated as follows:

(1) $$\left[\frac{\text{c}.\text{p}.\text{m}.\;\text{experimental}\;\text{release}-\text{c}.\text{p}.\text{m}.\;\text{SR}}{\text{c}.\text{p}.\text{m}.\;\text{maximum}\;\text{release}-\;\text{c}.\text{p}.\text{m}.\;\text{SR}}\right]\times 100.$$

Results are shown as the mean of at least three independent experiments. In all experiments, SR was <20% [28].

### 2.6. Blocking Experiments

Blocking antibodies were anti-NKG2A (CD159a, IgG2b, Z199 BeckmanCoulter) anti-LIR-1 (CD85j, 292319, IgG2b, R&D Systems) and anti-HLA-I (HP-1-F7, IgG1, Santa Cruz), which blocks HLA-A, -B, -C, -E, and -G engagement [10, 29, 30]. IgG1 (11711) and IgG2b (20116, both from R&D Systems) were used as isotype controls. F(ab′)_2_ fragments (Jackson ImmunoResearch) were used to prevent ADCC [27, 30]. Controls without F(ab′)_2_ are explicitly named. Toxicity of any of the used reagents was carefully ruled out (Figure 8).

### 2.7. Blocking of Effector Cells

CRA were performed as described above. Heat inactivated human serum (HS) was obtained after informed consent from healthy volunteers. NK-92 cells were preincubated in RPMI1640 with 1% PenStrep and 10% HS for 30 minutes and kept within the same medium during additional 30 minutes of incubation with mAb concentrations of 0.1, 1 and 10 *μ*g/mL, respectively. Target cells were prepared as described above. NK-92 cells were washed twice to avoid interactions of the mAb with the later on coincubated target cell line. Cells were adjusted for a fix E : T ratio of 1.25 : 1, using 5 × 10^3^ target cells/100 *μ*L as before. In some of the experiments, this step was followed by additional preincubation with 11 *μ*g/mL F(ab′)_2_ 15 minutes prior to coincubation of NK and tumor cells. In samples classified as “untreated,” no F(ab′)_2_ was used [30, 31]. SR was always <20% for all cell lines except from MOLP-8, which showed a constantly high SR up to 36% [28]. To rule out toxicity of the mAb, in four experiments NK-92 were radioactively labeled (51CrNK) and treated as the unlabeled cells in a parallel series to the blocking assays. 51CrNK showed SR < 6% for all conditions with a standard deviation (SD) < 3%. No differences related to parameters F(ab′)_2_, antibody or concentration could be detected within statistical analysis [30].

### 2.8. Blocking of Tumor Cells

Target cells were incubated with 1 *μ*g/mL of the respective mAb at a cell density of 5 × 10^4^ cells/mL. Procedure and incubation times were the same as described for NK cells. F(ab′)_2_ was used at a concentration of 1.7 *μ*g/mL for all samples, inclusively the “untreated” control. Tumor cell incubation with mAb led to SR of <25% for MOLP-8 and RPMI8226 and up to 11% for all other cells.

### 2.9. Statistics

To control for indirect effects, statistical interpretation was done by multivariate Analysis of Variance (ANOVA). In all calculations, specific lysis was defined as the dependent variable. Antibodies, concentrations of the mAb, the targets and the use or no use of F(ab′)_2_ were defined as independent variables. Wherever appropriate, interdependencies between the variables were taken into account. All calculations were done by SPSS (IBM SPSS Statistics Version 19, Release 19.0.0).

## 3. Results

### 3.1. Characterization of NK-92 and Tumor Cell Lines

**Figure 1:**
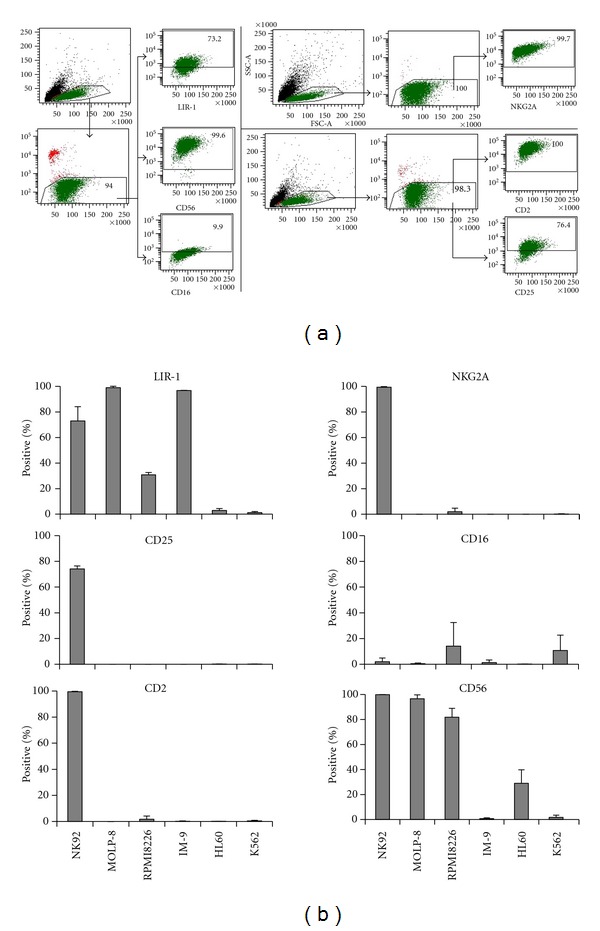
Expression of surface antigens. (a). Flow cytometric phenotyping of all cell lines was done with mABs against LIR-1, CD56, CD16, CNKG2A, CD2, and CD25 as shown for NK-92. (b). Surface antigen expression on NK-92 and tumor cell lines. Values given as % positive staining after subtraction of isotype control.

NK-92 was found to express high levels of LIR-1 based on flow cytometric analysis (Figure 1). We confirmed a high expression of NKG2A and CD25, as well as small amounts of Fc*γ*RIII (CD16) [10]. Hereby, but not regarding LIR-1 expression, NK-92 cells share important similarities with the CD56^bright^ subset of NK cells [32].

**Figure 2:**
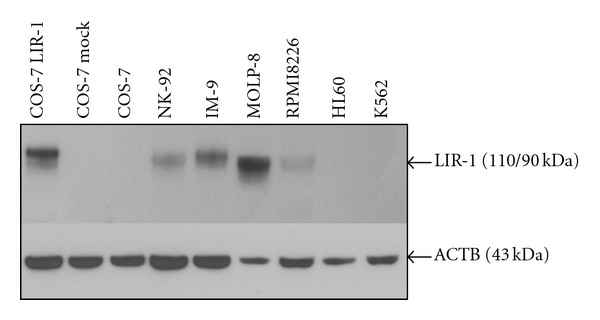
LIR-1 expression on NK-92 and target cells. Western blot analysis of LIR-1 expression on NK-92 cells and tumor cell lines. Untreated as well as LIR-1 or mock transfected COS-7 cells negative and positive controls, respectively. After film development, the membrane was stripped and reincubated with ACTB antibodies as a loading control. 4 *μ*g anti-LIR-1 and 10 *μ*g anti-ACTB (1 : 1000) were used for antigen detection. Lanes contended 35 *μ*g of total protein. As also shown by flow cytometric analysis, NK-92 as well as IM-9 and MOLP-8 expresses high levels of LIR-1, whereas no detection of LIR-1 was possible on HL60 and K562 cells.

**Figure 3:**
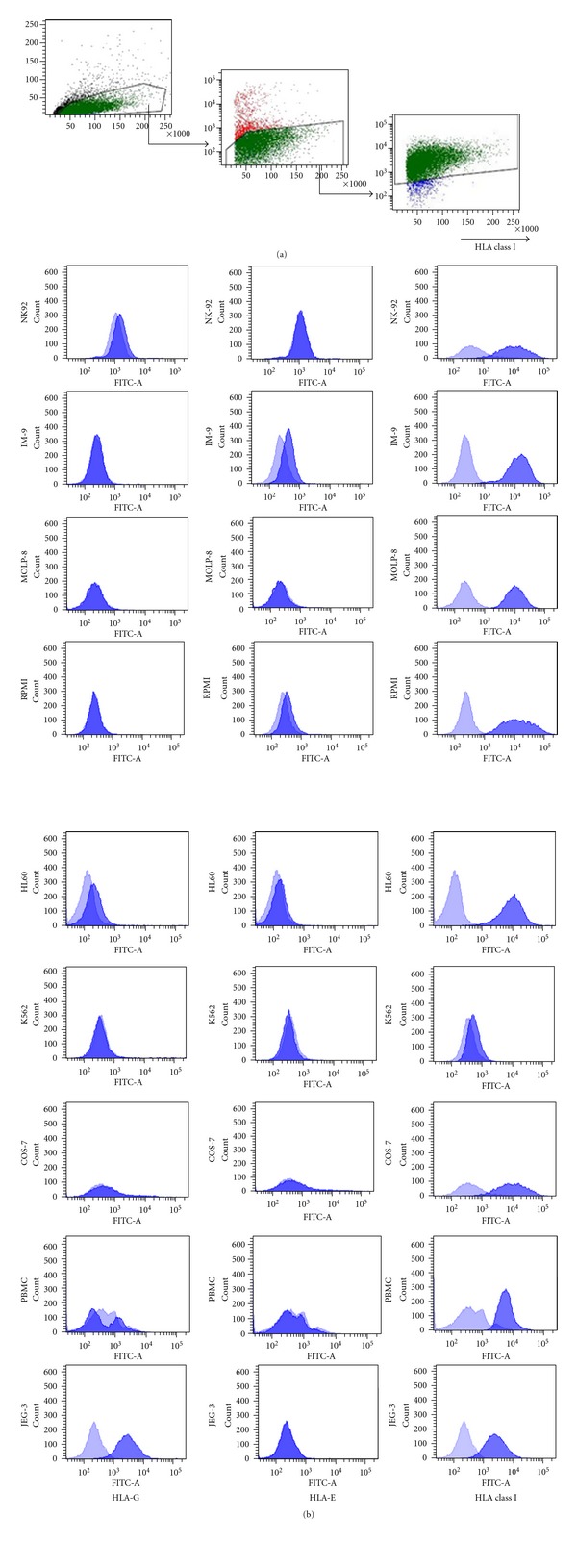
HLA expression. (a). Representative flow cytometric gating strategy for analysis of HLA expression on all used cell lines (shown for RPMI8226). All cells were stained with anti-HLA-I/-E/-G IgG1, followed by secondary goat anti-mouse IgG1 FITC. (b). HLA class I molecules were detectable on all cell lines except for negative control K562. JEG-3 were strongly positive for HLA-6, while detection on NK92 and HL60 was marginal. HLA-E as the ligand for NK62A lacked on all cell linesexcept on minor staining on IM-9.

LIR-1 was present on myeloma cells as well as on IM-9, but no target cell line expressed NKG2A. Western blot analysis confirmed the pattern of LIR-1 expression, and the strength of band representation reflected the staining intensity detected by flow cytometry (Figure 2). LIR-1 or mock-transfected as well as naïve COS-7 served as positive and negative controls, respectively. For evaluation of HLA class I expression, K562 served as negative controls. All cell lines except K562 were HLA class I positive which correlated with earlier investigations for MM (Figure 3) [13, 33]. LIR-1 has a broad spectrum of ligands, but its binding properties are weak. As HLA-G is the strongest binding partner, we evaluated HLA-G expression on all cell lines [4, 6, 34, 35]. Only JEG-3 were positive for HLA-G. Thus, interaction of LIR-1 in later-on conducted CRA was restricted to other binding partners.

The choice of NK-92 and those distinct tumor cell lines instead of primary cells allowed an isolated view on the inhibitory capacities of LIR-1. No increase of cytotoxicity due to blockade of NKG2A could be expected in subsequent blocking assays, for no target cell line expressed the only known NKG2A ligand HLA-E [36]. Furthermore, NK-92 has been described before to lack inhibitory KIR molecules [37, 38].

Other inhibitory LIRs that can be found on NK cells are only LIR-3 (ILT5) and LIR-8 as well as soluble LIR-4 for which the ligands are not yet detected [6].

LIR-1 could therefore be considered to be the only known major inhibitory receptor in this context and was expressed at high levels (Figures 1 and 2). Influence of so far unknown inhibitory receptors was ruled out by selective blockade of LIR-1.

Due to these findings, we considered the use of NK-92 and the chosen tumor cell lines as an ideal system to study the discrete influence of LIR-1 on modulation of NK-cell cytotoxicity.

### 3.2. Myeloma Cells Are Highly Susceptible to NK-92 Mediated Killing

**Figure 4:**
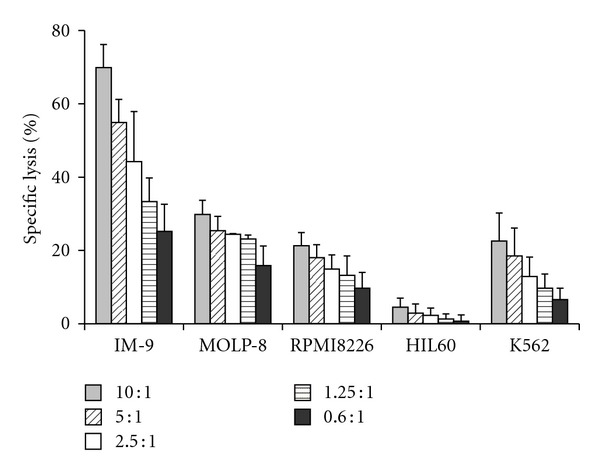
NK-92 mediated killing of target cell lines is dependent on applied E : T ratio. CRA revealed a clearly E : T-dependent level of NK-92 mediated cytotoxicity. Results of CRA are represented as means of at least three independent experiments ± SD. Values in the table below are given as % specific lysis. AML cell line HL60 was almost resistant to NK-92 mediated killing. NK-92 cells were stimulated with 200 U/ml IL-2 the day before the assay.

Cytotoxicity of NK-92 against a panel of tumor cell lines was investigated in CRA at different E : T ratios (Figure 4) [28]. MM cell lines and IM-9 were efficiently lysed by NK-92, with highest results for IM-9 (E : T 10 : 1; specific lysis 69.9 ± 6.3%) followed by MOLP-8 (29.8 ± 3.9%), K652 (22.6 ± 7.6%), and RPMI8226 (21.3 ± 3.6%). HL60 was almost resistant to lysis (4.5 ± 2.5%).

### 3.3. Blocking of LIR-1 on NK-92 Does Not Increase Target Cell Lysis

**Figure 5:**
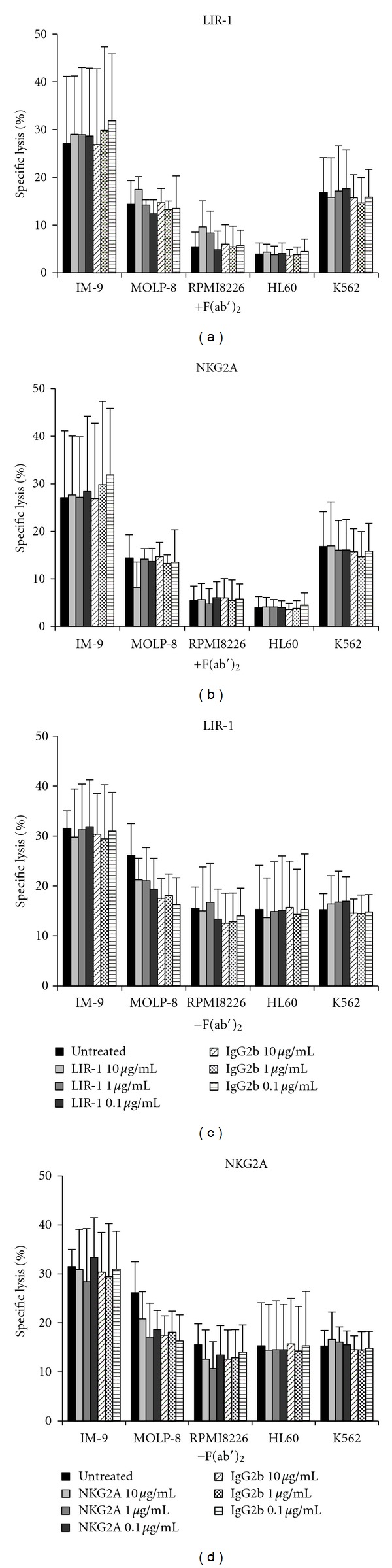
Blocking of CD85j and CD159a on NK-92 does not increase specific lysis. Blocking assays were performed as 4-hour CRA. NK-92 were preincubated with human serum and treated with the respective mAb (0.1, 1 and 10 *μ*g/mL). E : T ratio of 1.25 : 1 was used throughout all experiments with a total of 5 × 10^3^ target cells/200 *μ*l. Results are shown as means of triplicates of at least three independent experiments. (a/b): F(ab′)_2_ was added after the last washing step prior to coincubation with target cells and maintained within the medium throughout the whole experimental period. (C/D): Samples without F(ab′)_2_. In samples classified as “untreated”, neither F(ab′)_2_ nor Abs were used. SR was always <20% for all cell lines except from MOLP-8, which showed a constantly high SR up to 36%. Statistical analysis was performed by ANOVA and did not show any significant effect of Ab use.

To evaluate the influence of LIR-1 in myeloma cell lysis, mAbs were used to block LIR-1 receptor-ligand interactions. As target cells lacked the expression of the HLA-E molecule, blocking of NKG2A was not expected to alter the results but was conducted as a negative control. No significant increase of tumor cell lysis could be achieved by any of the mAbs despite high concentrations (Figure 5).

**Figure 6:**
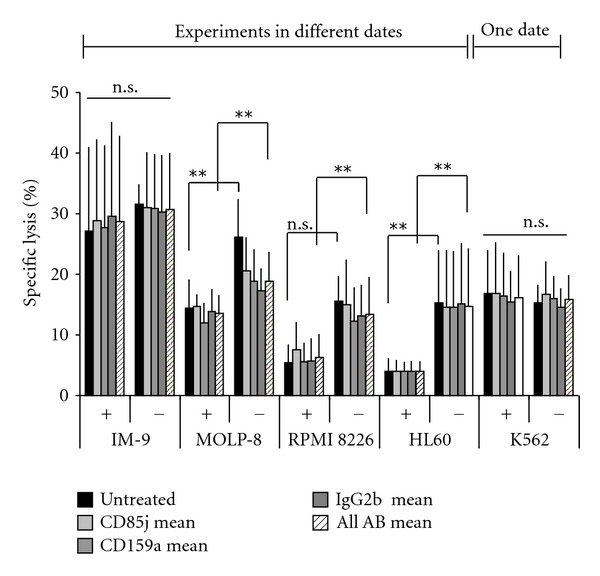
Use of F(ab′)_2_ does not significantly influence cytotoxicity of NK-92 cells. Statistical analysis (ANOVA) reveals a seemingly protective effect by F(ab′)_2_ fragments against NK-92 mediated lysis. As influence of the type of antibody and the used concentration were excluded, experimental data were merged. Results were highly significant (untreated ± F(ab′)_2_/Ab use ± F(ab′)_2_): IM-9 *P* = 0.317/*P* = 0.211; MOLP-8 *P* = 0.017/*P* = 0.002; RPMI8226 *P* = 0.052/*P* < 0.001; HL60 *P* = 0.009/*P* < 0.001; K562 *P* = 0.692/*P* = 0.771). The same settings were used throughout all experiments. In the experimental row, K652 tests were performed at the same day and within the same panel. For all other target cells, tests including F(ab′)_2_ were performed first and were followed by those without additional treatment 1 week later. Though IM-9 renders more susceptible to lysis to a nonsignificant degree, time is the factor most likely to be taken into account, for targets incubated with “untreated” NK cells showed the same changes in sensitivity towards lysis.

As specific lysis of target cells by NK-92 was found to be independent from mAb concentration and type, results are presented as means of the used concentrations (0.1/1/10 *μ*g/mL) or in a separate bar as means of concentration and mAbs (CD85j, CD159a, and IgG2b) (Figure 6). For MOLP-8, RPMI8226 and HL60, a significant influence of F(ab′)_2_ towards a decreased lysis seemed to be relevant, but it could not be taken into account.

What first might appear as a protective effect of the applied F(ab′)_2_ towards a reduced lysis of target cells could also be observed in the untreated sample and must therefore be considered to be a side effect caused by cell culture procedure. Only experiments with K562 were performed at the same day with and without F(ab′)_2_ and the observed specific lysis of K562 was the same for both experimental rows. This confirms the thesis of culture side effect to be responsible for significant changes in experimental results.

It is possible that LIR-1 influence could not be measured, if a maximum level of NK-activation had already been achieved before blocking of the inhibitory receptor [38, 39]. Interleukin (IL)-2 requirement during cell culture, induction of the high potential activating receptor NKp44 by IL-2 [40], and origin of NK-92 from rapidly progressive NK cell lymphoma [38, 41] favor a preactivated condition.

### 3.4. Blocking of Neither LIR-1 Nor HLA Class I on Target Cells Increases Target Cell Lysis

**Figure 7:**
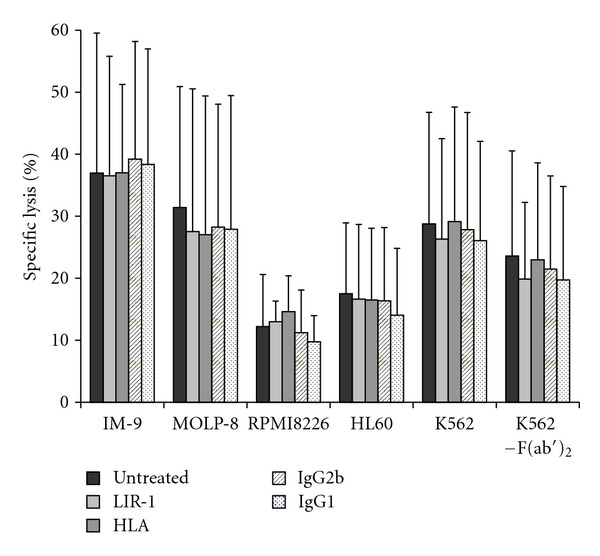
Blocking of LIR-1 and HLA on tumor cells does not lead to increased lysis by NK-92 cells. LIR-1 (CD85j) and HLA class I molecules of the tumor cell lines were blocked as before on the NK-92 cells. After incubation and extended washing, standard CRA were performed. Neither LIR-1 nor HLA blocking showed significant increase of tumor cell lysis. All experiments were performed with the use of F(ab′)_2_ to prevent ADCC. Statistical analysis performed by ANOVA excluded any significant effect of either isotype or Ab on target cell lysis, compared to untreated samples. Discrete investigation of K562 treatment, performed as a comparison of cumulated values for all Ab-incubated samples with or without F(ab′)_2_, excluded any influence of additional F(ab′)_2_ on the results (*P* = 0.218).

As expected, HLA-A, -B, -C, -E, and -G blockade on tumor cells did not show any influence on lysis (Figure 7) since blocking of LIR-1 as the only relevant inhibitory NK cell receptor on NK-92 had already not modulated cytotoxicity. We also decided to selectively block LIR-1 receptors on the tumor cells before coincubation with NK-92. Though not being likely to directly change NK-cell properties, LIR-1 expression might contribute to MM resistance in so far unknown ways as its role in immune regulations is still fairly unknown (discussed below). As LIR-1 surface expression increases during B-cell and DC maturation [42], it might be directly involved in cell-cell interactions that promote survival and growth. In our experiments, LIR-1 expression on target cells seemed not responsible for a resistance to lysis (Figure 7).

### 3.5. mAb or F(ab′)_2_ Have No Toxic Effects on NK-92 under Experimental Conditions

**Figure 8:**
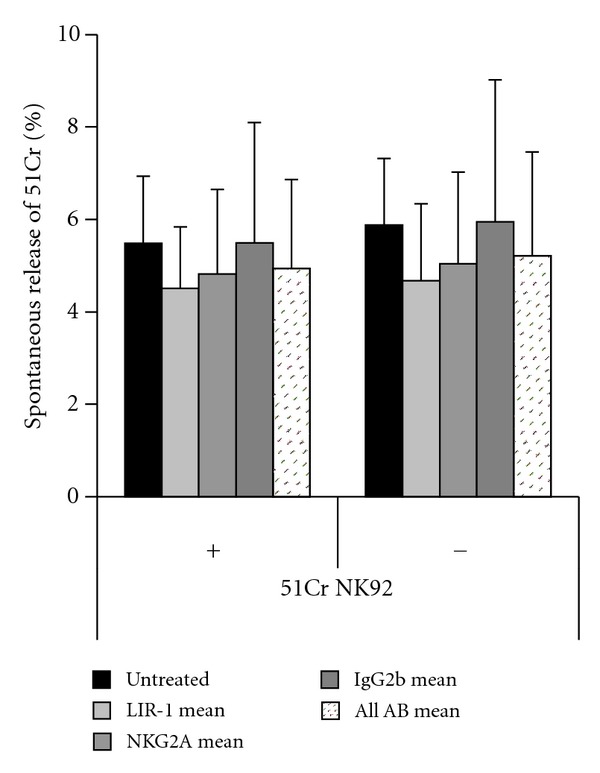
Neither antibodies nor F(ab′)_2_ show toxic effects on NK-92 cells. NK-92 were labeled with 51Cr and incubated with Abs against LIR-1, NKG2A, or IgG2b. Isotype control with (+) or without (−) F(ab′)_2_ is under the same conditions as the unlabeled effector cells in the blocking experiments. SR is given as mean of four independent experiments for untreated NK cells and as mean of an experimental series with concentration of 10, 1 and 0.1 *μ*g/mL. “All Ab mean” integrates those results. F(ab′)_2_ concentration was 11 *μ*/mL. Spontaneous release was <6%. As this control panel was performed in parallel to regular NK-92 blocking experiments, interferences by reagents, temperature, incubation period, cell viability, or technical equipment can be excluded.

To rule out toxic effects of mAb, NK-92 were labeled with 51Cr and incubated with the respective mAb in parallel to the conducted experiments. In four independent experiments, no harmful effect of anti-LIR-1, anti-NKG2A or F(ab′)_2_ could be observed (Figure 8).

### 3.6. F(ab′)_2_ Stabilize Pattern of Tumor Lysis

**Figure 9:**
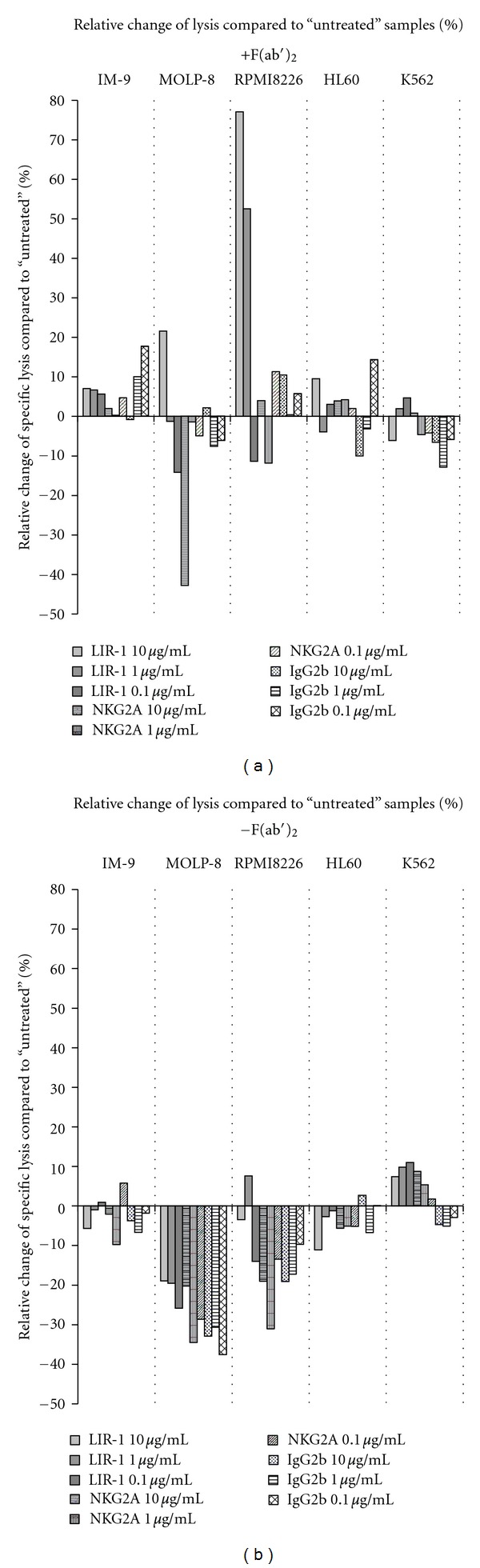
Use of F(ab′)_2_ after blocking NK-92 receptors seems to balance lysis pattern. Values show the % relative increase or decrease of target cells lysis in a standard 4-hour CRA, calculated as follows: % relative change of specific lysis = [(% specific lysis “antibody”) − (% specific lysis “untreated”)]/[% specific lysis “untreated”]. Though no concentration of the used mAbs showed a significant change in tumor lysis, interesting differences in the resulting lysis-patterns could be detected. (a) Apart from outliers, use of F(ab′)_2 _ evens out mAb effects in the blocking experiments, leading to values that oscillate close to the origin in both directions. (b) Sparing F(ab′)_2_ decreases specific lysis, predominantly for MOLP-8 and RPMI8226 cells.

Although not significantly affecting tumor lysis, there seemed to be an important influence of the F(ab′)_2_ fragments (Figure 9). They stabilized the results even though prior evaluation of surface molecules did only show very low amounts of CD16 (see above). Apart from outliers, use of F(ab′)_2_ seemed to even out mAb effects in the blocking experiments, leading to values that oscillate close to the origin in both directions (a). Sparing those fragments decreased relative lysis, predominantly relevant for MOLP-8 and RPMI8226 cells. Only K562 cells rendered more susceptible to NK mediated lysis with a lack of F(ab′)_2_ though not at a significant level (b).

## 4. Discussion

The aim of the present investigations was to evaluate if LIR-1 on NK cells inhibits NK-92 mediated cytotoxicity against different tumor cell lines. Secondly, presence of LIR-1 on the target cells was validated concerning its influence NK cell mediated lysis. LIR-1 was assumed to be the only relevant inhibitory receptor on NK-92 as stated above (characterization of NK-92 and tumor cell lines) [38]. Surprisingly, no inhibitory influence of LIR-1 on NK-92 within cytotoxicity assays against different tumor cell lines could be detected after treatment with specific antibodies against LIR-1 (Figures 5–7).

By now, involvement of LIR-1 in protecting the fetus from abortion has become common immunological knowledge, and so has the adoption of this mechanism by tumor cells by expressing the LIR-1 ligand HLA-G [43–45]. Furthermore, viruses express highly affinitive LIR-1 ligands for protection against immune defense and LIR-1 serves as a receptor for bacterial detection [5, 8]. Data about the specific role of this receptor in “normal” action of lymphocytes, especially NK cells, are more conflicting and rare. While LIR-1 inhibition of T cell and monocyte activation has been shown before, only a few studies focus on NK cell modulation, and even less refer to experimental settings in which HLA-G was not present on the target cell [46–48]. Available publications emphasize data that confirm an important inhibitory effect of LIR-1, but a more questioning view might refer to the number of experiments mentioned that did not deliver the pronounced outcome. We will give a short overview of available functional studies of LIR-1 on donor-derived NK cells and NK cell lines in order to demonstrate the conflicting knowledge that is available.

Godal et al. made an attempt in revealing the influence of LIR-1 on dNK in cytotoxicity against HLA-G negative AML and ALL blasts. Their results indicate that LIR-1 does only serve as a weak inhibitory NK cell receptor in the absence of HLA-G on tumor cells, but might be relevant in situations with low KIR expression as seen within the first months after stem cell transplantation (SCT) [10].

Other authors used merely HLA-transfected 721.221, murine cells (P815) or immature dendritic cells (iDC) as target cells, but often had the benefit of comparing results from NK cell lines to the performance of NK cells derived from healthy donors. In 2008, Yawata et al. conducted a series of degranulation assays of dNK against HLA class I deficient 721.221 in order to investigate the involvement of distinct receptors in “missing-self” recognition and were not able to identify any involvement of LIR-1 [49]. More successful LIR-1 mediated inhibition was achieved by Morel and Bellon who tested the cytotoxicity of dNK and NK-92 against HLA-G and -B transfected 721.221 in CRA with subsequent use of blocking antibodies [39]. Their prestudies found only 15% of LIR-1 positive dNK to be stimulated by target contact and these responsive dNK were chosen for the subsequent studies. Comparative analysis of NK-92 and dNK in different types of assays were conflicting.

No such conflict was observed by Favier et al., who incubated dNK and the NK cell line NKL with 721.221-G1 and used blocking Abs against LIR-1 and HLA-G [50]. The same was true for Vitale et al. They incubated dNK with different HLA transfected cell lines in standard CRA and increased lysis by LIR-1 blockade, but unfortunately, no explicit prevention of antibody-dependent cellular cytotoxicity (ADCC) was mentioned [51]. At last, Colonna et al. showed the expected LIR-1 influence in different experimental setups with NKL, serotonin releasing RBL cells, as well as dNK and donor derived LIR+ T cells (dTK) [21]. Interestingly, in all assays with either dTK or dNK against 721.221-B*2705, anti-LIR-1 could only partially revert transfection-induced inhibition, indicating either incomplete binding of anti-LIR-1 to the receptor or the presence of other receptors apart from LIR-1 that bind to the ligand.

Moreover, in reverse ADCC (rADCC) assays with dNK against P815, only few clones were inhibited by LIR-1. Here again, NK cell clones have shown to exhibit less predictable outcomes than cell lines [21].

Summarizing the available data, NK cell lines seem to be more reliable than dNK concerning LIR-1 mediated downregulation of cytotoxicity. The results for polyclonal dNK show high variances between the different clones that are mostly not characterized in detail. Successful LIR-1 mediated inhibition by HLA-transfected 721.221 has abundantly been shown. Involvement of additional undetected receptors could not always be excluded, and different types of assays performed with the same effector and target cells could lead to highly diverging results. Investigations of cytotoxicity of dNK and donor-derived tumor cells are rare and no sufficient information is available about the role of LIR-1 in the absence of HLA-G. Current opinion about the inhibitory influence of LIR-1 is predominantly based on investigations at the feto-maternal interface or has been gained from settings in which only a single HLA molecule was present on the target cells—mostly HLA-G or -B on 721.221. The performed experiments do not sufficiently cover the extensive binding capacities of LIR-1 to HLA class I.

Although we are aware of problems concerning the comparability of NK cell lines to donor-derived NK cells (dNK), we have chosen an experimental setting that allows studying isolated influence of LIR-1 on cytotoxicity. Our major goal was to provide a model system to overcome the common practice of using transfected target cell lines in cytotoxicity assays. Being aware of the necessity of future efforts to confirm the present findings in a brighter panel of cells and cell lines, these results might provide a first step towards a new understanding of LIR-1.

Available data do not sufficiently support the direct implication of LIR-1 in NK cell inhibition. Upregulation of the receptor does not necessarily favor immunosuppression but might correlate with the acquisition of memory. LIR-1 surface expression increases during B cell and DC maturation [42] as well as during cytomegalovirus infection [52] and acquisition of T-cell memory [53, 54]. We suppose that an increasing LIR-1 expression also correlates with the acquisition of NK cell memory [55], supported by the surveillance that a high LIR-1 level on NK cells leads to an effective lysis of HIV-infected DC [56].

LIR-1 seems to have a high impact on regulating the balance between activation and inhibition during immune responses [42]. It might do so by cooperation with other receptors like KIRs, which favor a clustering of MHC-I [57]. As HLA-G is the only LIR-1 ligand that generates covalent dimers and trimers at the cell surface, aggregation might be a precondition to receptor's activation. It has been proposed that these complexes increase the avidity towards LIR-1, explaining the discrepancy between the relatively low affinity of LIR-1 to its ligand and the high relevance within the context of fetal tolerance [58]. Homo- or heterotypic complex formation of the LIR-1 receptor and its ligands might be a key factor that regulates the degree of NK cell inhibition. By this, LIR-1 might work as a rheostat of NK cell activation. This hypothesis would fit the capability of LIR-1 to sense the overall HLA class I expression on human tissues. An increase during aging as well as memory acquisition could elevate the necessary threshold for activation during a parallel process of increase of activating NK cell receptors caused by repeated pathogen contacts.

## 5. Conclusions

Within the present study, no alteration of NK mediated cytotoxicity against MM was observed after blockade of LIR-1. Being the only functional inhibitory receptor within this setting, major known side effects by, for example, KIR have been ruled out. This unexpected outcome opens the door to fruitful discussions about the complexity of LIR-1 interactions and its potential role within tumor defense. It is very likely that the present presumptions about functions of LIR-1 within immune regulation are far behind the real impact. We hypothesize that LIR-1 has a key role as a rheostat of NK cell modulation and is strongly involved in the acquisition of NK cell memory.

## References

[1] Herberman RB, Nunn ME, Lavrin DH (1975). Natural cytotoxic reactivity of mouse lymphoid cells against syngeneic and allogeneic tumors. I. Distribution of reactivity and specificity. *International Journal of Cancer*.

[2] Lanier LL (2005). NK cell recognition. *Annual Review of Immunology*.

[3] Ferlazzo G, Tsang ML, Moretta L, Melioli G, Steinman RM, Münz C (2002). Human dendritic cells activate resting natural killer (NK) cells and are recognized via the NKp30 receptor by activated NK cells. *Journal of Experimental Medicine*.

[4] Chapman TL, Heikema AP, West AP, Bjorkman PJ (2000). Crystal structure and ligand binding properties of the D1D2 region of the inhibitory receptor LIR-1(ILT2). *Immunity*.

[5] Nakayama M, Underhill DM, Petersen TW (2007). Paired Ig-like receptors bind to bacteria and shape TLR-mediated cytokine production. *Journal of Immunology*.

[6] Borges L, Cosman D (2000). LIRs/ILTs/MIRs, inhibitory and stimulatory Ig-superfamily receptors expressed in myeloid and lymphoid cells. *Cytokine and Growth Factor Reviews*.

[7] Samaridis J, Colonna M (1997). Cloning of novel immunoglobulin superfamily receptors expressed on human myeloid and lymphoid cells: structural evidence for new stimulatory and inhibitory pathways. *European Journal of Immunology*.

[8] Cosman D, Fanger N, Borges L (1997). A novel immunoglobulin superfamily receptor for cellular and viral MHC class I molecules. *Immunity*.

[9] Borges L, Hsu ML, Fanger N, Kubin M, Cosman D (1997). A family of human lymphoid and myeloid Ig-like receptors, some of which bind to MHC class I molecules. *Journal of Immunology*.

[10] Godal R, Bachanova V, Gleason M (2010). Natural killer cell killing of acute myelogenous leukemia and acute lymphoblastic leukemia blasts by killer cell immunoglobulin-like receptor-negative natural killer cells after NKG2A and LIR-1 blockade. *Biology of Blood and Marrow Transplantation*.

[11] Beck S, Barrell BG (1988). Human cytomegalovirus encodes a glycoprotein homologous to MHC class-I antigens. *Nature*.

[12] Fahnestock ML, Johnson JL, Renny Feldman RM, Neveu JM, Lane WS, Bjorkman PJ (1995). The MHC class I homolog encoded by human cytomegalovirus binds endogenous peptides. *Immunity*.

[13] Carbone E, Neri P, Mesuraca M (2005). HLA class I, NKG2D, and natural cytotoxicity receptors regulate multiple myeloma cell recognition by natural killer cells. *Blood*.

[14] Cook G, Campbell JDM (1999). Immune regulation in multiple myeloma: the host-tumour conflict. *Blood Reviews*.

[15] Palumbo A, Anderson K (2011). Multiple myeloma. *The New England Journal of Medicine*.

[16] Porrata LF, Litzow MR, Markovic SN (2001). Immune reconstitution after autologous hematopoietic stem cell transplantation. *Mayo Clinic Proceedings*.

[17] Ault KA, Antin JH, Ginsburg D (1985). Phenotype of recovering lymphoid cell populations after marrow transplantation. *Journal of Experimental Medicine*.

[18] Chalifour A, Jeannin P, Gauchat J-F (2004). Direct bacterial protein PAMP recognition by human NK cells involves TLRs and triggers *α*-defensin production. *Blood*.

[19] Mandelboim O, Lieberman N, Lev M (2001). Recognition of haemagglutinins on virus-infected cells by NKp46 activates lysis by human NK cells. *Nature*.

[20] Nguyen S, Dhedin N, Vernant J-P (2005). NK-cell reconstitution after haploidentical hematopoietic stem-cell transplantations: immaturity of NK cells and inhibitory effect of NKG2 A override GvL effect. *Blood*.

[21] Colonna M, Navarro F, Bellon T (1997). A common inhibitory receptor for major histocompatibility complex class I molecules on human lymphoid and myelomonocytic cells. *Journal of Experimental Medicine*.

[22] Carretero M, Cantoni C, Bellón T (1997). The CD94 and NKG2-A c-type lectins covalently assemble to form a natural killer cell inhibitory receptor for HLA class I molecules. *European Journal of Immunology*.

[23] Burnette WN (1981). ‘Western Blotting’: electrophoretic transfer of proteins from sodium dodecyl sulfate-polyacrylamide gels to unmodified nitrocellulose and radiographic detection with antibody and radioiodinated protein A. *Analytical Biochemistry*.

[24] Riteau B, Rouas-Freiss N, Menier C, Paul P, Dausset J, Carosella ED (2001). HLA-G2, -G3, and -G4 isoforms expressed as nonmature cell surface glycoproteins inhibit NK and antigen-specific CTL cytolysis. *Journal of Immunology*.

[25] Goodman HS (1961). A general method for the quantitation of immune cytolysis. *Nature*.

[26] Newman W (1982). Selective blockade of human killer cells by a monoclonal antibody. *Proceedings of the National Academy of Sciences of the United States of America*.

[27] Le Gal FA, Riteau B, Sedlik C (1999). HLA-G-mediated inhibition of antigen-specific cytotoxic T lymphocytes. *International Immunology*.

[28] Whiteside TL, Bryant J, Day R, Herberman RB (1990). Natural killer cytotoxicity in the diagnosis of immune dysfunction: criteria for a reproducible assay. *Journal of Clinical Laboratory Analysis*.

[29] Bryceson YT, Ljunggren H-G, Long EO (2009). Minimal requirement for induction of natural cytotoxicity and intersection of activation signals by inhibitory receptors. *Blood*.

[30] Riteau B, Menier C, Khalil-Daher I (2001). HLA-G1 co-expression boosts the HLA class I-mediated NK lysis inhibition. *International Immunology*.

[31] Rouas-Freiss N, Marchal RE, Kirszenbaum M, Dausset J, Carosella ED (1997). The alpha 1 domain of HLA-G1 and HLA-G2 inhibits cytotoxicity induced by natural killer cells: is HLA-G the public ligand for natural killer cell inhibitory receptors?. *Proceedings of the National Academy of Sciences of the United States of America*.

[32] Cooper MA, Fehniger TA, Caligiuri MA (2001). The biology of human natural killer-cell subsets. *Trends in Immunology*.

[33] Palmisano GL, Contardi E, Morabito A, Gargaglione V, Ferrara GB, Pistillo MP (2005). HLA-E surface expression is independent of the availability of HLA class I signal sequence-derived peptides in human tumor cell lines. *Human Immunology*.

[34] Chapman TL, Heikema AP, Bjorkman PJ (1999). The inhibitory receptor LIR-1 uses a common binding interaction to recognize class I MHC molecules and the viral homolog UL18. *Immunity*.

[35] Shiroishi M, Tsumoto K, Amano K (2003). Human inhibitory receptors Ig-like transcript 2 (ILT2) and ILT_4_ compete with CD8 for MHC class I binding and bind preferentially to HLA-G. *Proceedings of the National Academy of Sciences of the United States of America*.

[36] Braud VM, Allan DSJ, O’Callaghan CA (1998). HLA-E binds to natural killer cell receptors CD94/NKG2A, B and C. *Nature*.

[37] Davidson CL, Li NL, Burshtyn DN (2010). LILRB1 polymorphism and surface phenotypes of natural killer cells. *Human Immunology*.

[38] Maki G, Klingemann H-G, Martinson JA, Tam YK (2001). Factors regulating the cytotoxic activity of the human natural killer cell line, NK-92. *Journal of Hematotherapy and Stem Cell Research*.

[39] Morel E, Bellón T (2008). HLA class I molecules regulate IFN-*γ* production induced in NK cells by target cells, viral products, or immature dendritic cells through the inhibitory receptor ILT2/CD85j. *Journal of Immunology*.

[40] Vitale M, Bottino C, Sivori S (1998). NKp44, a novel triggering surface molecule specifically expressed by activated natural killer cells, is involved in non-major histocompatibility complex-restricted tumor cell lysis. *Journal of Experimental Medicine*.

[41] Gong JH, Maki G, Klingemann H-G (1994). Characterization of a human cell line (NK-92) with phenotypical and functional characteristics of activated natural killer cells. *Leukemia*.

[42] Young NT, Waller ECP, Patel R, Roghanian A, Austyn JM, Trowsdale J (2008). The inhibitory receptor LILRB1 modulates the differentiation and regulatory potential of human dendritic cells. *Blood*.

[43] Ponte M, Cantoni C, Biassoni R (1999). Inhibitory receptors sensing HLA-G1 molecules in pregnancy: decidua-associated natural killer cells express LIR-1 and CD94/NKG2A and acquire p49, an HLA-G1-specific receptor. *Proceedings of the National Academy of Sciences of the United States of America*.

[44] Paul P, Rouas-Freiss N, Khalil-Daher I (1998). HLA-G expression in melanoma: a way for tumor cells to escape from immunosurveillance. *Proceedings of the National Academy of Sciences of the United States of America*.

[45] Sheu J, Shih I-M (2010). HLA-G and immune evasion in cancer cells. *Journal of the Formosan Medical Association*.

[46] Saverino D, Fabbi M, Ghiotto F (2000). The CD85/LIR-1/ILT2 inhibitory receptor is expressed by all human T lymphocytes and down-regulates their functions. *Journal of Immunology*.

[47] Dietrich J, Cella M, Colonna M (2001). Ig-like transcript 2 (ILT2)/leukocyte Ig-like receptor 1 (LIR1) inhibits TCR signaling and actin cytoskeleton reorganization. *Journal of Immunology*.

[48] Fanger NA, Cosman D, Peterson L, Braddy SC, Maliszewski CR, Borges L (1998). The MHC class I binding proteins LIR-1 and LIR-2 inhibit Fc receptor-mediated signaling in monocytes. *European Journal of Immunology*.

[49] Yawata M, Yawata N, Draghi M, Partheniou F, Little A-M, Parham P (2008). MHC class I specific inhibitory receptors and their ligands structure diverse human NK-cell repertoires toward a balance of missing self-response. *Blood*.

[50] Favier B, LeMaoult J, Lesport E, Carosella ED (2010). ILT2/HLA-G interaction impairs NK-cell functions through the inhibition of the late but not the early events of the NK-cell activating synapse. *FASEB Journal*.

[51] Vitale M, Castriconi R, Parolini S (1999). The leukocyte Ig-like receptor (LIR)_−1_ for the cytomegalovivus UL18 protein displays a broad specificity for different HLA class I alleles: analysis of LIR_−1_ NK cell clones. *International Immunology*.

[52] Berg L, Riise GC, Cosman D (2003). LIR-1 expression on lymphocytes, and cytomegalovirus disease in lung-transplant recipients. *The Lancet*.

[53] Young NT, Uhrberg M, Phillips JH, Lanier LL, Parham P (2001). Differential expression of leukocyte receptor complex-encoded Ig-like receptors correlates with the transition from effector to memory CTL. *Journal of Immunology*.

[54] Vivier E, Anfossi N (2004). Inhibitory NK-cell receptors on T cells: witness of the past, actors of the future. *Nature Reviews Immunology*.

[55] Sun JC, Beilke JN, Lanier LL (2009). Adaptive immune features of natural killer cells. *Nature*.

[56] Scott-Algara D, Arnold V, Didier C (2008). The CD85j^+^ NK cell subset potently controls HIV-1 replication in autologous dendritic cells. *PLoS ONE*.

[57] Kirwan SE, Burshtyn DN (2005). Killer cell Ig-like receptor-dependent signaling by Ig-like transcript 2 (ILT2/CD85j/LILRB1/LIR-1). *Journal of Immunology*.

[58] Gonen-Gross T, Achdout H, Gazit R (2003). Complexes of HLA-G protein on the cell surface are important for leukocyte Ig-like receptor-1 function. *Journal of Immunology*.

